# Bilinguals implicitly name objects in both their languages: an ERP study

**DOI:** 10.3389/fpsyg.2014.01415

**Published:** 2014-12-09

**Authors:** Katie Von Holzen, Nivedita Mani

**Affiliations:** ^1^Laboratoire Psychologie de la Perception UMR 8158, Université Paris DescartesParis, France; ^2^Psychology of Language Research Group, Georg-Elias-Müller Institute of Psychology, University of GöttingenGöttingen, Germany

**Keywords:** bilingualism, implicit naming, phonological priming, lexical access, ERP

## Abstract

Upon being presented with a familiar name-known image, monolingual infants and adults implicitly generate the image's label (Meyer et al., 2007; Mani and Plunkett, 2010, 2011; Mani et al., 2012a). Although the cross-linguistic influences on overt bilingual production are well studied (for a summary see Colomé and Miozzo, 2010), evidence that bilinguals implicitly generate the label for familiar objects in both languages remains mixed. For example, bilinguals implicitly generate picture labels in both of their languages, but only when tested in L2 and not L1 (Wu and Thierry, 2011) or when immersed in their L2 (Spivey and Marian, 1999; Marian and Spivey, 2003a,b) but not when immersed in their L1 (Weber and Cutler, 2004). The current study tests whether bilinguals implicitly generate picture labels in both of their languages when tested in their L1 with a cross-modal ERP priming paradigm. The results extend previous findings by showing that not just do bilinguals implicitly generate the labels for visually fixated images in both of their languages when immersed in their L1, but also that these implicitly generated labels in one language can prime recognition of subsequently presented auditory targets across languages (i.e., L2–L1). The current study provides support for cascaded models of lexical access during speech production, as well as a new priming paradigm for the study of bilingual language processing.

## Introduction

Research on speech production has awarded considerable attention to the stages involved in a speaker's selection of an appropriate lexical item(s) to communicate her message. Among other issues, this work has examined how a speaker selects one word among other appropriate partially activated words for production, whether these other activated words interact with the speakers' choice and production of the chosen word, and the extent to which the phonological and semantic features of these other activated words are retrieved during speech production. Most models of speech production agree that the search for the appropriate lexical item in production also lends activation to items semantically related to the chosen word, either through activation of semantic features shared by the words or through activation of the corresponding lexical nodes of the semantically related words (Dell, 1986; Levelt, 1989; Roelofs, 1992; Caramazza, 1997). Models of speech production disagree, however, with regard to the extent to which the phonological features associated with these competing lexical nodes are retrieved in speaking. Discrete models of speech production suggest that while semantically related lexical nodes are simultaneously activated, phonological activation is restricted to the selected lexical node alone (Levelt, 1989; Levelt et al., 1999). In contrast, cascaded models of lexical access assume that the phonological properties of semantically related lexical nodes are all simultaneously activated (Dell, 1986; Caramazza, 1997; Dell et al., 1997).

Particularly useful for resolving the discrepancies between cascaded and discrete models is the study of bilingual speech production. Between their two languages, bilinguals have many translation equivalents with varying levels of phonological overlap. One class of words, cognates, contain similar orthographic-phonological forms across languages. If, as argued by discrete models of speech production, only the phonological information for the corresponding node is activated, bilingual speech production should be similar for both cognate and non-cognate words. Studies investigating bilingual cognate and non-cognate picture naming, however, demonstrate a difference in naming latency between cognates and non-cognates (Costa et al., 2000; Hoshino and Kroll, 2008; Colomé and Miozzo, 2010; Strijkers et al., 2010; Poarch and van Hell, 2012). These results have overwhelmingly demonstrated that bilinguals activate phonological information from the non-target language, providing support for cascaded models of lexical access by showing that both selected and non-selected lexical nodes activate their corresponding phonological codes. Due to their special status across languages, however, the presence of cognate words may induce a bilingual processing mode (Wu and Thierry, 2010b). Stronger support for cascaded models of lexical access is therefore provided by studies not examining cognate word stimuli, yet still show that phonological information from both languages is activated during production in one language (Hermans et al., 1998; Colomé, 2001; Kaushanskaya and Marian, 2007; Hoshino and Thierry, 2011; Wu and Thierry, 2011). For example, Spalek and colleagues (Spalek et al., 2014) had German-English bilinguals produce adjective-noun pairs that either contained (e.g., *green goat*) or did not contain (e.g., *green skirt*) overt phonological onset overlap in English. Some trials, however, although they did not overlap in English, did contain phonological onset overlap once translated to German (e.g., *blue flower*, “blaue Blume”). Trials that overlapped overtly in English and covertly in German modulated the ERP (event-related potential) response in comparison to non-related trials, suggesting that German translation equivalents of the English words were simultaneously activated and influenced production, despite the entire experiment being conducted in English.

In the current study, we examine a special instance of the retrieval of phonological and semantic information in the selection of lexical nodes for production. Unlike the body of research described previously, which focused on overt production, we turn to covert production, or implicit label generation. This allows for the study of the information bilinguals use to name, or activate upon visual fixation of, objects, before information is ultimately chosen for production. Specifically, we examine whether bilingual speakers implicitly produce the labels of visually fixated images and whether they do so in both their languages.

Upon viewing an image, studies suggest that the label for this image is implicitly generated, and that this implicitly generated label can prime recognition of a subsequently presented, related target. In Meyer et al.'s study (Meyer et al., 2007; see also Jescheniak et al., 2002; Meyer and Damian, 2007), for example, adults were presented with an unlabeled prime image (i.e., *boy*) followed by a visual display of four images, one of which was a homophone of the unlabeled picture prime (i.e., *buoy*). There was no semantic overlap between the homophone target and the prime image. Indeed, the only overlap between the two images lies in the labels for the two images—any preference for looking toward the homophone image could, therefore only be explained as a result of participants' implicitly generating the label for both images and this implicitly generated label subsequently priming recognition of the related target image. Indeed, consistent with this explanation, participants were more likely to fixate the phonologically related target image compared to phonologically unrelated distractor images. This finding has been taken to show that participants implicitly generate the labels for visually fixated images, i.e., that they retrieve the phonological properties associated with the labels for visually fixated images. Such implicit label generation has also been found in infants using phonologically related prime-target pictures (Mani and Plunkett, 2010, 2011; Mani et al., 2012a), suggesting that auditory and visual information are integrated at as young as 18-months-of-age.

Implicit label generation has also been explored using the ERP method in a cross-modal priming paradigm. Desroches et al. (2009) presented participants with picture prime—spoken target word pairs that were either identical, onset-overlapping, rhymes, or unrelated, while simultaneously measuring participants' ERP responses to the spoken target words (see Mani et al., 2012b for similar studies with infants). ERPs (event-related potentials) are averaged waveforms of electrical brain activity (EEG) time-locked to the presentation of stimuli and can provide a measure of speech processing with a high temporal resolution of brain activity. One ERP component investigated by Desroches et al. (2009), the N400 (Kutas and Hillyard, 1984), described as a negative inflection peaking at approximately 400 ms after stimulus onset, indexes the integration of a stimulus into the context set by a preceding stimulus: the larger the N400 amplitude, the more difficult the integration process between stimuli. Although Desroches et al., reported some variation in component latency, N400 amplitude was reduced for both onset overlapping and rhyming prime-target pairs. Using a cross-modal priming procedure, the authors argued, ensures that any priming effects were the result of top-down processes resulting from connections at the phonological and lexical levels instead of bottom-up influence due to acoustic overlap between prime and target. This conclusion also assumes that participants implicitly generated the label for the picture primes, which ultimately primed recognition of the spoken target word.

Unlike monolingual speakers, however, bilingual speakers have at least two labels for every object, one in one language (e. g. *dog*, English) and one in the other (e. g. *Hund*, German). When viewing objects, therefore, bilinguals may implicitly generate the label in one or both of their languages. In terms of overt speech production in bilinguals, cross-language effects have been found when participants are tested in both their L1 and L2 and immersed in their L1 (Costa et al., 2000; Colomé, 2001; Hoshino and Kroll, 2008; Colomé and Miozzo, 2010; Strijkers et al., 2010; Poarch and van Hell, 2012) or their L2 (Hermans et al., 1998; Costa et al., 2000, 2009; Kaushanskaya and Marian, 2007; Hoshino and Thierry, 2011; Spalek et al., 2014). Explicit naming or overt production may tap into different processes compared to implicit naming or covert production, particularly to do with the later stages of the speech monitoring process involved in overt production and due to delays introduced by the actual production of muscle movements (see Wu and Thierry, 2011 for similar suggestions) which may allow the required time for effects of L2 access to appear. It is, therefore, important to distinguish between findings of studies examining explicit and implicit naming. With regard to covert production, however, some evidence suggests that bilinguals may implicitly generate the labels for objects in both their languages, but that this depends on the language of testing, as well as whether they are immersed in their L1 or L2 (see Wu and Thierry, 2010b for a discussion of context in bilingual experiments): Previous studies have demonstrated that bilinguals implicitly label objects in both languages when they are immersed in their L2 and tested in L2, but results differ when participants are tested in their L1 (Spivey and Marian, 1999; Marian and Spivey, 2003a,b; Wu and Thierry, 2011). In the current study, using the cross-modal priming paradigm of Desroches et al. (2009), we examine whether bilinguals implicitly generate the label for objects in both of their languages when they are tested in their L1 and immersed in an L1 environment, a context that has previously failed to yield this effect (Weber and Cutler, 2004).

For bilinguals immersed in an L2 environment, successful L2 performance may come at the cost of L1 fluency. Linck et al. (2009) compared English learners of Spanish who were either immersed in a Spanish, L2 environment, or remained in their native, L1 English environment. When tested on both comprehension and production, an interesting asymmetry appeared between the two groups of participants: although the L2 performance was better for learners immersed in an L2 environment than their L1 environment counterparts, these participants showed decreased L1 access. This pattern of results led the authors to suggest that when immersed in their L2, the learners inhibited activation of their L1. Within a group of participants tested before and after L2 immersion, however, Baus et al. (2013) found similar results, although only for low frequency, non-cognate words, suggesting that the decrease in L1 access during L2 immersion is the result of decreased L1 usage and not L1 inhibition. Although the purpose of the current paper is not to resolve which mechanisms are at work during L2 or L1 immersion, these studies highlight the effects that immersion can have on L1 and L2 access and performance.

To our knowledge, only one study has specifically investigated the question of whether bilinguals implicitly generate the label in one or both of their languages (although others have indirectly addressed it, see below). Wu and Thierry (2011) presented Chinese-English participants with pairs of pictures and asked them to judge whether the labels of the two pictures rhymed in L2 English (Experiment 1) or shared a character in L1 Chinese (Experiment 2). The stimuli were manipulated such that some of the picture pairs overlapped in one language (e. g. rhymed in L2 English), while others overlapped in the other language (e. g. character overlap in L1 Chinese). EEG data was recorded throughout the experiment to examine the neurocognitive indices of cross-language lexical access. Consistent with the standard N400 priming effect (Kutas and Hillyard, 1984), when asked to evaluate L1 Chinese overlap, participants found picture pairs whose labels overlapped in Chinese easier to process relative to unrelated picture pairs. Similarly, when asked to evaluate L2 English overlap, participants found picture pairs whose labels rhymed in English easier to process relative to unrelated pictures pairs whose labels did not rhyme. Critically, when asked to evaluate L2 English overlap, picture pairs whose labels overlapped in L1 Chinese were also easier to process, suggesting that Chinese-English bilinguals activated both the L1 and L2 labels for the pictures. However, an effect of L2 English overlap was not found when participants were making rhyme judgments in L1 Chinese. Wu and Thierry attribute this asymmetric effect to the possibility that L2 word forms are not implicitly generated while making judgments in L1. In contrast, L1 word forms were activated during L2 processing and Wu and Thierry suggest that this is the result of bilinguals' inability to prevent interference from their L1 during L2 speech planning (Green, 1998).

Experiments using the visual world paradigm, however, suggest that both languages are activated even when bilingual participants are tested in their L1 and immersed in an L2 environment. In a series of experiments, Marian and Spivey (2003a,b) and Spivey and Marian (1999) presented Russian-English bilinguals with a visual display containing several objects. In one version of the experiment, participants were instructed in L1 Russian to move a target object (e. g. *marka*, “stamp”). Although they were tested in Russian, participants were more likely to look at a distractor object that had a phonologically related label in L2 English (e.g., *flomaster*, “marker”) than a distractor object with an unrelated label (e.g., *lineka*, “ruler”). The results suggest that the word form of objects were also activated in L2 English, causing the English label for the phonologically related distractor object (i.e., *flomaster*, “marker”) to compete for activation with the Russian label for the target object (i.e., *marka*, “stamp”). In another version of the experiment, where participants were instructed in L2 English, they were more likely to look at a target that was phonologically related in L1 Russian, than a distractor object with an unrelated label. In other words, bilinguals implicitly generated the label for the objects in both of their languages, regardless of the language of testing.

Nevertheless, participants in the Marian and Spivey experiments were immersed in their L2, where they heard their L2 every day in their surrounding environment. Using a similar paradigm to that of Marian and Spivey, Weber and Cutler (2004) extended the results of Marian and Spivey to participants tested in their L2 while immersed in their L1. Interestingly, Weber and Cutler did not find evidence of L2 activation when participants were tested in their L1 while immersed in an L1 environment. Weber and Cutler suggest that these results may reflect a difference in the background of their participants and the testing environment in comparison to the bilinguals tested by Spivey and Marian: The bilinguals tested by Spivey and Marian were immersed in their L2, English, perhaps increasing the likelihood that English would be co-activated. In contrast, the Dutch-English bilinguals tested by Weber and Cutler lived in the Netherlands and used their L1, Dutch, in their everyday life, making L2 English less relevant for activation when participants were tested in L1. It may, therefore, be more likely for L2 words to be activated during L1 processing when the participants are immersed in their L2.

It is of interest, however, that the Chinese-English bilinguals tested by Wu and Thierry (2011) do not show effects of L2 activation in L1 processing, despite being immersed in their L2 (similar to the language environments of Spivey and Marian, 1999; Marian and Spivey, 2003a,b). A potential explanation for this difference might come from nature of the task performed by participants. Spivey and Marian did not explicitly ask participants to judge the phonological overlap between target and distractor object labels in either of the languages of the participants, while Wu and Thierry focused participants' attention on phonological overlap for the picture pairs in one language, i.e., either their L1 or their L2. It is possible that this conscious focus on phonological overlap in the one language may reduce the influence of the “other” language, especially when the other language is the less dominant L2. The current study, therefore, does not focus participants' attention on phonological overlap in either of their languages. Instead, we asked participants to perform a non-linguistic task (a picture matching task), which drew their attention away from the relationship between the prime and target. We suggest that this provides a more accurate measure of whether bilinguals implicitly generate the labels of visually presented images in both their languages by not biasing their attention to linguistic relationships.

Using a cross-modal priming paradigm, participants were presented with visual picture primes (presented in silence) followed by auditory L1 targets. Although it differs from the picture-picture task of Wu and Thierry, it is similar to the visual world paradigm (Spivey and Marian, 1999; Marian and Spivey, 2003a,b; Weber and Cutler, 2004) where the target is a spoken word. This paradigm, shown to elicit an N400 component for both onset and rhyme related picture prime—target word pairs (Desroches et al., 2009), allows not only for the study of implicit label generation, but also removes the potential role of acoustic overlap between prime and target. This also allowed for an unbiased investigation of cross-linguistic priming on auditory word recognition (i.e., L2 picture prime label—L1 auditory target). Although studies that have investigated auditory word recognition in bilinguals are increasing (Sinai and Pratt, 2002; Ju and Luce, 2004; Weber and Paris, 2004; Blumenfeld and Marian, 2005, 2007; Cutler et al., 2006; Marian et al., 2008; Rueschemeyer et al., 2008; Canseco-Gonzalez et al., 2010; FitzPatrick and Indefrey, 2010; Altvater-Mackensen and Mani, 2011; Lagrou et al., 2011; see also Shook and Marian, 2012; Weber and Broersma, 2012; Von Holzen and Mani, 2012; FitzPatrick and Indefrey, 2014), there are relatively few studies that have specifically investigated whether cross-linguistic priming can influence auditory word recognition (Phillips et al., 2006; Pratt et al., 2013). These studies, however, used both languages within their experiment, possibly creating an artificial bilingual environment (Grosjean, 1997). In the current study, we address this problem by testing participants exclusively in their L1.

**Figure 1:**
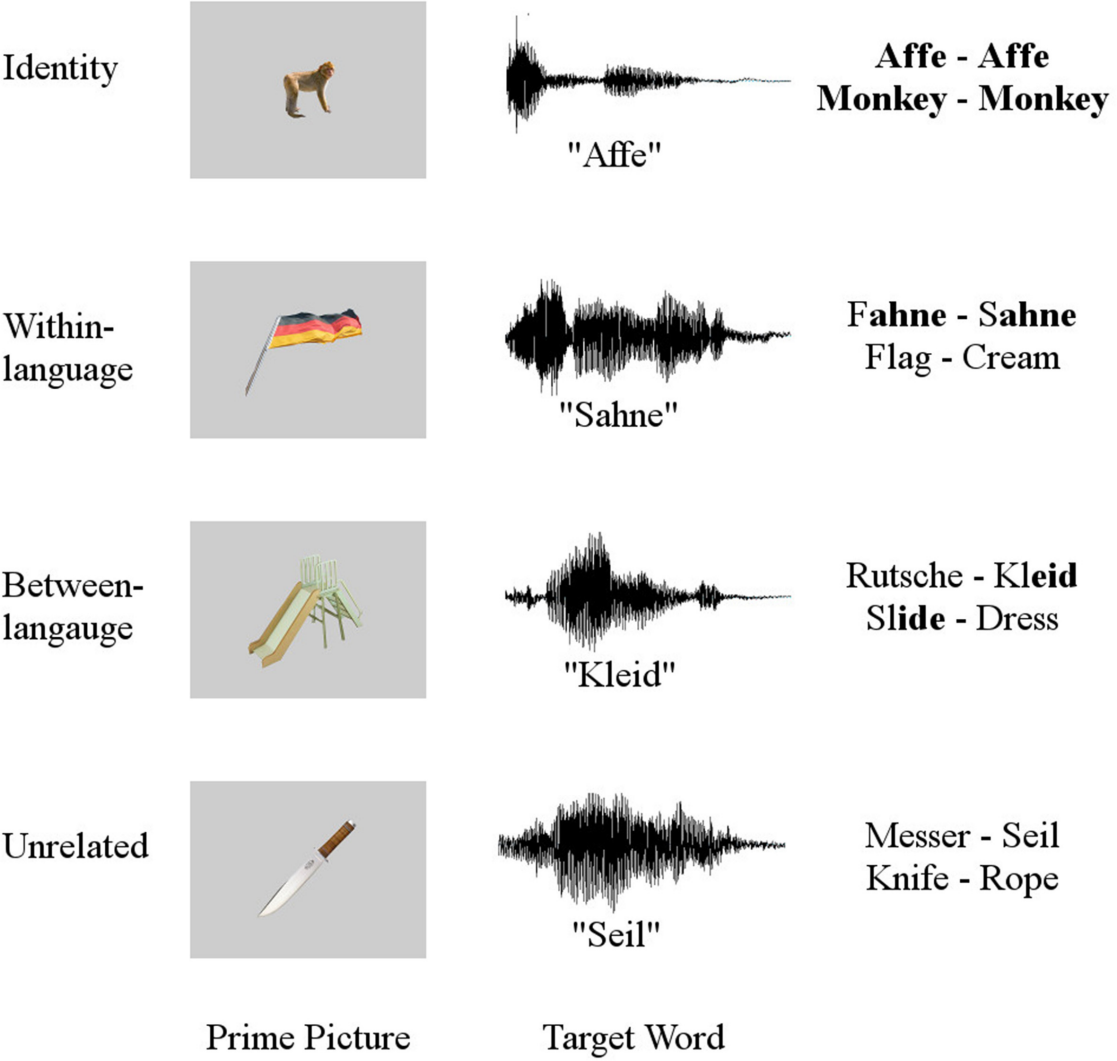
**Examples of stimuli used in each condition**. A prime picture, its corresponding German target word, as well as the English and German translations of these stimuli are given.

In the current study, implicit generation of both language labels was examined by manipulating the relationship between the L1 and L2 labels for the picture prime and the L1 auditory target words. Thus, we included four conditions in the experiment: (1) picture prime labels and L1 target words were identical in L1 German; (2) L1 German labels for the picture primes and L1 target words were phonologically related in German; (3) L2 English labels for the picture primes and L1 target words sounded similar^1^; (4) L1 and L2 labels for the picture prime and L1 target words were phonologically, orthographically, or semantically unrelated (see Figure 1 for examples). Similar to Wu and Thierry (2011), in trials where picture prime labels and L1 target words were related within- or between-language, the relationship was offset-overlap, which has already been demonstrated to elicit a N400 priming effect in a similar study with monolingual participants (Desroches et al., 2009). In summary, the current study aims to answer two questions:

1. When viewing images, do bilinguals implicitly generate the labels for these images in one or both of their languages?
2. Can implicitly generated labels in L2 influence processing of auditory words in L1?

Our index of these effects is obtained from Event-related potential (ERP) recordings of participants' brain activity as they heard the auditory target labels, focusing on the N400 component. In the study of bilingualism, the N400 waveform has been used as a measure of priming between prime-target pairs that are related across languages semantically (Alvarez et al., 2003; Phillips et al., 2004; Martin et al., 2009; Palmer et al., 2010), orthographically (De Bruijn et al., 2001), phonologically (Altvater-Mackensen and Mani, 2011), as well as through their translations (Thierry and Wu, 2007; Wu and Thierry, 2010a, 2011; see Moreno et al., 2008 for a review of ERP use in bilingualism). We suggest that, in contrast to the visual world paradigm, participants' ERPs may be a more sensitive index of the subtle differences involved in bilingual language processing (Mueller, 2005), given that participant eye gaze may be influenced by the number of objects in the visual display (Sorensen and Bailey, 2007) or simply not reflect competition effects (Dahan and Tanenhaus, 2004). In combination with the cross-modal priming paradigm, the use of ERPs may help clarify the differences in L2 activation during L1 processing when bilinguals are immersed in their L2 (Spivey and Marian, 1999; Marian and Spivey, 2003a,b) or their L1 (Weber and Cutler, 2004).

If participants were to implicitly generate the labels for the picture primes in their L1, this would be reflected by a significant reduction in N400 amplitude when the target word is either the same as the L1 label for the picture prime or phonologically related to the L1 label for the picture prime. Crucially, if bilinguals also implicitly generate the L2 labels for the picture primes, then N400 amplitude should also be reduced when the target word sounds similar to the L2 label for the picture prime. Furthermore, our manipulations allow for a a comparison of priming effects for prime-target pairs that are related within- (e.g., L1 to L1) and between-languages (e.g., L2 to L1). This relates to a question within the study of bilingualism which asks whether a bilinguals' two languages are organized into two separate, but connected lexicons or are integrated into one large lexicon and would present evidence of cross-language priming in an experiment conducted entirely in one language.

## Materials and methods

### Participants

The current study tested a group of 16 German-English bilinguals (age *M* = 27.63; age *SD* = 7.82; age range = 20−48). Participants were recruited as bilinguals from the local population in a middle-sized German city and told that the purpose of the experiment was to examine their visual perception and were afterwards debriefed on the full purpose of the experiment. After the experiment, participants filled out a language proficiency questionnaire (adapted from Rueschemeyer et al., 2008). In this questionnaire, participants indicated the age at which they began learning both German and English. All participants had been exposed to both German and English before age 10. Participants also indicated their proficiency in reading, writing, listening, speaking, and syntax in German and English. These proficiency scores were averaged to create a combined proficiency score for both German and English. Participants reported an average combined proficiency score that was similar in both German (*M* = 9.19; *SD* = 1.02) and English (*M* = 9.05; *SD* = 0.99; *p* > 0.05). In addition, participants also took part in a picture-naming task where we could assess the accuracy with which they labeled images in German and English. The results of these tests are reported in the Results section, showing that participants correctly and equally quickly labeled images in both German and English. All but three participants reported German to be their mother tongue and English their second language. Of these three, two participants reported German and English to be their mother tongue while one participant reported English to be her mother tongue, having learned German before she was 3 years of age. Therefore, we consider German to be the L1 of the participants and English, their L2, although these two languages are balanced. Participants were living in Germany at the point of testing, immersed in their L1. Before the experiment participants signed an informed consent form approved by the ethics committee of the University Göttingen and received 15 Euros afterwards for their participation.

### Stimuli

The stimuli consisted of 120 primes and 120 targets, resulting in 120 prime-target pairs. Primes were visually presented in silence, i.e., were presented as unlabeled, familiar images. Targets were presented auditorily, i.e., the picture prime was followed by an auditorily presented target word. A female, native speaker of German recorded all target words. The relationship between the labels for the prime image and the auditory target labels was manipulated to create four conditions: identity—picture prime labels and L1 target words were identical in German (e. g., prime picture *monkey* “Affe”—target word *Affe*), within-language condition—L1 German labels for the picture primes and L1 target words were phonologically related in German (e. g., prime picture *flag* “Fahne”—target word *Sahne* “cream”), between-language condition—L2 English labels for the picture primes and L1 target words sounded similar (e. g., prime picture *slide* “Rutsche”—target word *Kleid* “dress”), or unrelated—L1 and L2 labels for the picture prime and L1 target words were phonologically, orthographically, or semantically unrelated (e. g., prime picture *knife* “Messer”—target word *Seil* “rope”). The 120 prime-target pairs were distributed across the four conditions with 30 pairs per condition. Prime and target words across languages were matched on the frequency of the words as well as the number of syllables and phonemes in the words (*p*'s > 0.05). Figure 1 contains example stimuli from each condition. A list of stimuli can be found in the Supplementary Material.

### Procedure

#### Main experiment

Participants were seated in a dimly lit, quiet experimental room, facing a 92 cm wide and 50 cm high TV screen at a distance of 100 cm from the screen. All instructions given to participants, including the written instructions presented on an instruction sheet, were in German. Participants were presented with 120 trials distributed across the four conditions, with 30 trials per condition. Each trial began with a fixation cross displayed in the center of the screen for 1000 ms. Following the offset of the fixation cross, participants were presented with the prime image centrally located on the screen. The prime image remained on screen for 500 ms (from 1000 to 1500 ms into the trial) followed by a blank, black screen. At 1550 ms into the trial, 50 ms after the offset of the prime picture, participants were presented with an auditory target word. At 3000 ms (i.e., 1450 ms after the onset of the target label), a second image was displayed that was either identical to the prime image or was different to the prime image. This image remained on-screen for 500 ms (from 3000 to 3500 ms into the trial) and was followed by a blank, black screen for a further 1000 ms (from 3500 to 4500 ms into the trial). Participants were instructed to indicate in this interval (from 3500 to 4500 ms into the trial) whether the second image matched the first image presented or not, by pressing one of two buttons in front of them. Participants were informed that the experiment investigated the mechanisms underlying their perception of the similarity between the two images presented. They were informed that they would hear spoken words during the experiment but that their task was to ignore these spoken words. This was done in order to avoid any overt attention to the relationship between the labels for the prime images and the target words.

#### Production task

Following the main experiment, participants also completed a production task, where they were asked to label a series of 60 images aloud in both German and English. Half of the participants labeled the images in German first, while the other half labeled in English first. Stimuli used in the production task were the prime images from the within- and between-language conditions. The answers provided by the participants were automatically recorded via a microphone, time-locked to the appearance of the image on-screen. Production data was analyzed offline to determine whether participants labeled the prime images from these conditions with the label we had chosen for each picture. If the label provided by a participant was different from the chosen label for an image, then the trial containing this image was removed from the main experiment. This was to ensure that the labels implicitly generated by individual participants were related to the target words in the two critical conditions. For example, in the between-language condition, the picture prime “beagle” could also be given the label “dog.” However, the label “dog” does not sound similar to the target word “Igel” and therefore no longer fulfills the between-language manipulation. For the between-language condition, this removed 13% of trials (71 trials) and for the within-language condition 10.34% of trials (60 trials)^2^.

### EEG recording and analysis

Electrophysiological data was recorded using the Biosemi Active Two Amplifier system at a sampling rate of 2048 Hz from 32 Ag/AgCl electrodes placed according to the 10–20 convention. Electrode offsets were kept at less than 25 μ V. Electroencephalogram was re-referenced offline to the averaged mastoid reference. EEG data was then filtered off-line using a 0.1 Hz high-pass forward filter and a 20 Hz low-pass, zero-phase shift filter.

Averaging and artifact rejection were carried out using the BESA software (Version 5.3). Blink and movement artifacts were automatically rejected using a 100 Hz amplitude cut-off across all electrodes. Epochs were defined from −200 to 1000 ms from the onset of the auditory target word. We then analyzed the data in 50 ms time windows (from 0 to 1000 ms) to determine the onset and offset of significant differences between conditions. Based on this analysis, and the known onset of the N400 (Kutas and Hillyard, 1984), we focused our analyses on the time window between 300 and 400 ms (the N400 window; Desroches et al., 2009).

Average ERP waveforms were quantified by computing mean amplitudes per subject, electrode and condition in the selected time windows. ERP waveforms were baseline corrected by subtracting the mean amplitude for the baseline time window (-200 to 0 ms) from the selected time window. For purposes of data reduction, a selection of electrode locations was entered into data analysis, 16 electrodes divided into four regions and four lateral columns: frontal (F7, F3, F4, F8), fronto-central (FC5, FC1, FC2, FC6), central (T7, C3, C4, T8), and centro-parietal (CP5, CP1, CP2, CP6). Our analysis was based on specific planned comparisons between related conditions (identical, within- and between-language conditions) and the unrelated condition instead of a general condition effect; we therefore do not report the omnibus ANOVA (see Abelson and Prentice, 1997). Factors included in the repeated measures ANOVA were region (frontal, fronto-central, central, and centro-parietal), electrode laterality (4), and condition (2; related, unrelated).

## Results

**Figure 2:**
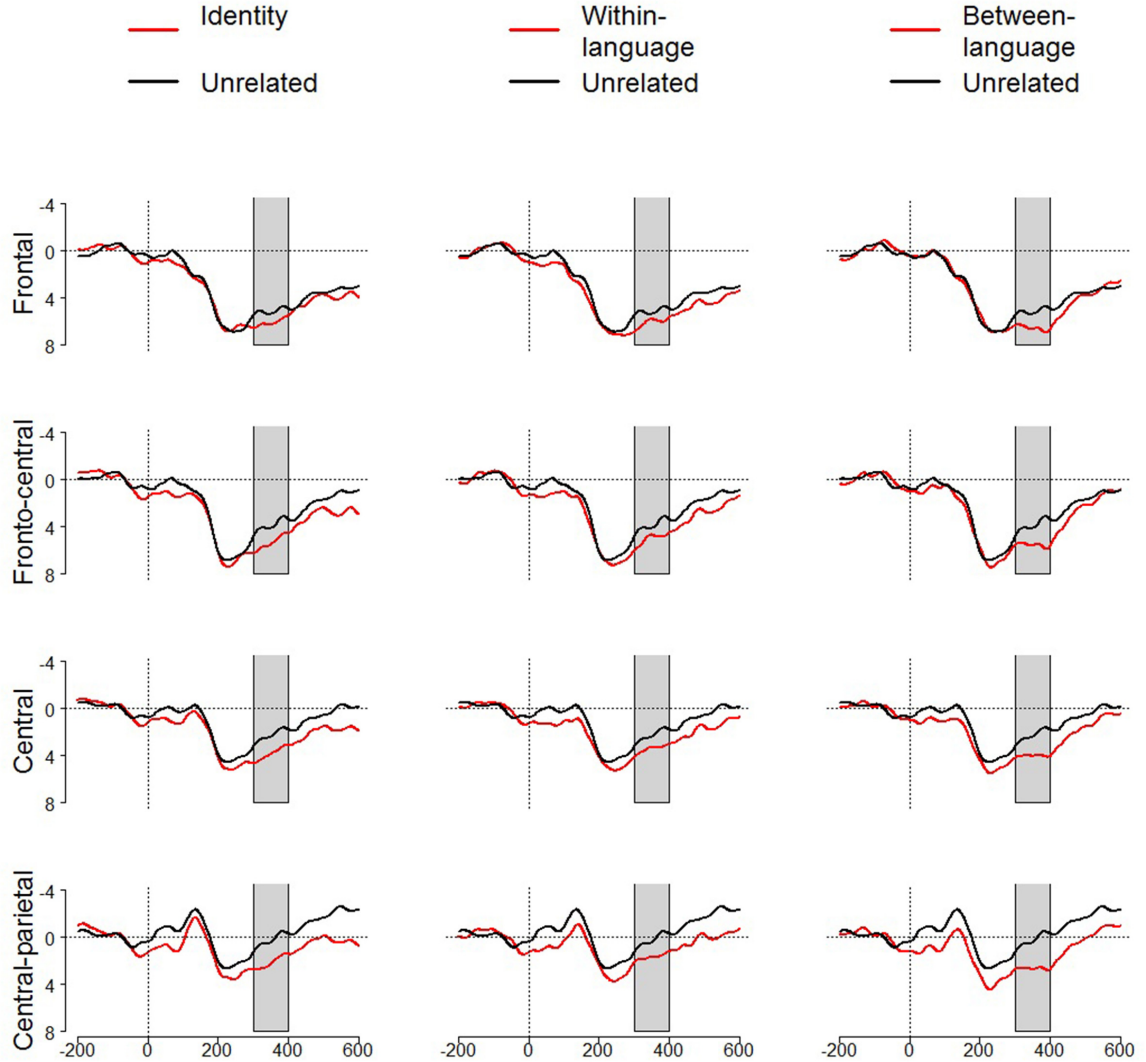
**Event-related potentials (ERPs) for each related (identity, within-language, between-language) and the unrelated condition comparison**. Graphs present data averaged across frontal, fronto-central, central, and central-parietal regions from −200 to 600 ms from the onset of the L1 target word (N400 window—300 to 400 ms—shaded in gray).

Figure 2 displays ERP waveforms aggregated across electrodes, separated by region (frontal, fronto-central, central, centro-parietal) for each of the three condition comparisons (identity vs. unrelated/within-language vs. unrelated/between-language vs. unrelated). We first examined the difference in the mean N400 amplitude of the brain potentials following identity and unrelated prime-target pairs. Here, a significant main effect of condition revealed that mean N400 amplitude was reduced for identity prime-target pairs relative to unrelated prime-target pairs, *F*_(1, 15)_ = 6.20, *p* = 0.025, η^2^_*p*_ = 0.29. No other interactions with condition were significant (*p*s > 0.25). Planned *post-hoc* analyses revealed that mean N400 amplitude for identity prime-target pairs was reduced relative to unrelated prime-target pairs across all regions, i.e., at frontal, *t*_(15)_ = 2.23, *p* = 0.042, *d* = 0.25, fronto-central, *t*_(15)_ = 2.95, *p* = 0.01, *d* = 0.37, and central *t*_(15)_ = 2.46, *p* = 0.026, *d* = 0.43, regions, and approached significance for the centro-parietal region, *t*_(15)_ = 1.92, *p* = 0.074, *d* = 0.48. As expected, complete match between the label for the prime image and the target word in the identity condition resulted in easier processing of the target word, relative to when the prime label was unrelated to the target word.

Next, we examined the difference in mean N400 amplitude for within-language related prime-target pairs and unrelated prime-target pairs. A repeated-measures ANOVA revealed a significant main effect of condition, *F*_(1, 15)_ = 4.54, *p* = 0.05, η^2^_*p*_ = 0.23, suggesting less negative N400 amplitude to L1 targets preceded by primes whose L1 labels were phonologically related to the L1 target words, relative to targets preceded by unrelated primes. No other interactions with condition were significant (*p*'s > 0.56). Planned *post-hoc* analyses revealed that mean N400 amplitude for within-language prime-target pairs was reduced, relative to unrelated prime-target pairs, across all regions, significant at frontal, *t*_(15)_ = 2.16, *p* = 0.047, *d* = 0.23, and fronto-central, *t*_(15)_ = 2.25, *p* = 0.04, *d* = 0.24, regions and approached significance at the centro-parietal region, *t*_(15)_ = 1.83, *p* = 0.088, *d* = 0.32, but not the central region (*p* > 0.12). In line with predictions, participants implicitly generated the label for the prime image in their L1, which speeded processing of the phonologically related L1 target word.

Finally, we examined the difference in mean N400 amplitude for between-language related prime-target pairs and unrelated prime-target pairs. An ANOVA comparing N400 amplitude across between-language related pairs and unrelated prime-target pairs revealed a near-significant main effect of condition, *F*_(1, 15)_ = 4.25, *p* = 0.057, η^2^_*p*_ = 0.22. No other interactions with condition were significant (*p*'s > 0.18). Planned *post-hoc* analyses revealed that mean N400 amplitude for between-language prime-target pairs was reduced, relative to unrelated prime-target pairs, across all regions, significant at central, *t*_(15)_ = 2.37, *p* = 0.032, *d* = 0.40, and centro-parietal, *t*_(15)_ = 2.13, *p* = 0.05, *d* = 0.53, regions, but not frontal or fronto-cental regions (*p'*s > 0.12). The only way that the prime image in between-language related prime-target pairs could influence recognition of the target, would be if participants were to *also* implicitly generate the label for the prime image in their L2, and for this implicitly generated L2 label to speed processing of the phonologically related L1 target word.

To examine whether there was any differences in the magnitude of the priming effect across related conditions, further analyses compared ERPs to targets between the related conditions. ANOVAs comparing N400 amplitude for identity and within-language, identity and between-language, and within- and between-language prime-target pairs revealed no significant main effect of condition (*p* > 0.38) or interactions with condition (*p*s > 0.15), except for a significant interaction between condition X electrode laterality for the comparison between the identity and between-language conditions, *F*_(3, 45)_ = 3.76, *p* = 0.031, η^2^_*p*_ = 0.20. Paired-samples *t*-tests, however, revealed that there was no significant difference between the identity and between-language conditions at each of the lateral columns (*p*'s > 0.3). The lack of a significant difference between the related conditions suggests a similar magnitude in the priming effect independent of a within- or between-language relationship between prime labels and target words.

In addition, we also measured the accuracy with which participants labeled the within and between-language related primes in German and English as well as the latency to name the images. In this task, conducted after the main experiment, participants were asked to overtly name the prime pictures from the within- and between-language conditions in German and English. Trials with no response were considered technical errors (German: 1.67%; English: 3.13%) and not included in the analysis. We considered responses correct if they accurately labeled the picture, regardless of the label we chose for the picture (e.g., *cat* or *kitty* for the prime picture *kitten*). Participants gave an incorrect response for 2.50% of trials in German and 2.70% of trials in English. Trials containing images whose labels participants incorrectly labeled were excluded from the analysis. There was no difference in accuracy for participants when they labeled pictures in German (96.83%) or English (94.17%; *p* >.3). In addition, there was no difference in reaction time for pictures named in German (*M* = 712.20 ms; *SD* = 188.24 ms) and English (*M* = 728.00 ms; *SD* = 185.05 ms; *p* > 0.75). Taken together, participants showed no difference between German and English in the production task.

## Discussion

The current study asked whether bilingual adults implicitly generate the label for words in one or both of their languages, and whether implicitly generated L2 labels can prime L1 auditory words. These findings suggest that bilingual adults implicitly generate the label for visually fixated images in both of their languages, and that this implicitly generated label can, subsequently influence recognition of an auditorily presented, similar sounding L1 target word. These results significantly extend previous findings to strongly support suggestions that (a) implicit generation of the labels of visually fixated images in both languages of bilinguals immersed in their L1 environment and (b) L2 prime labels influencing recognition of L1 target words, despite the experiment being carried out in an L1 environment with only L1 stimuli being used in the experiment. This presents a robust test of the extent to which bilinguals implicitly generate picture labels in their L2 as well as their L1 in an experiment situation that discourages such activation.

### Picture labels are implicitly generated in L1 and L2

Prime-target pairs whose labels were either identical (identity; i.e., prime picture *monkey* “Affe”—target word *Affe*) or phonologically related within L1 (within-language; i.e., prime picture *flag* “Fahne”—target word *Sahne* “cream”) elicited a reduction in N400 amplitude, suggesting that participants implicitly generated the label for prime pictures in their L1. That is, the implicitly generated L1 label subsequently primed the L1 target word as a result of the complete or phonological overlap between prime and target. This replicates previous studies with monolingual adults (Meyer et al., 2007; Desroches et al., 2009) and infants (Mani and Plunkett, 2010, 2011) that show that prime pictures presented in silence activate their labels and corresponding phonological information, priming subsequently presented identical or phonologically-related targets.

Critical to the current study's research questions, the reduction in N400 amplitude for L1 target words preceded by prime pictures whose label in L2 English was similar sounding to the L1 target word (between-language; i.e., prime picture *slide* “Rutsche”—target word *Kleid* “dress”), suggests that bilingual participants also implicitly generated the prime picture label in their L2. This demonstrates that bilinguals implicitly generate the labels for objects in not one, but both of their languages. In a previous study, Wu and Thierry (2011) asked Chinese-English bilinguals to preform rhyme judgments on picture pairs, some of which were rhyme pairs in either Chinese or English. Priming effects were elicited for rhyme pairs in both languages when participants were tested in L2 English, but when participants were tested in L1 Chinese, only Chinese rhyme pairs elicited priming effects. The results of Wu and Thierry suggest that whether participants implicitly generated picture labels in one or both languages, therefore, depended on the language they were tested in. Wu and Thierry conclude that this asymmetry shows that spoken language planning (i.e., implicit label generation) in L1 proceeds without activating L2 word information, but that bilinguals are unable to prevent L1 interference during L2 speech planning.

In the current study, however, we find that when participants are tested in their L1, they implicitly generate the label not only in L1, as Wu and Thierry found, but also in L2. We suggest that the difference between the current study and that of Wu and Thierry is the result of the tasks which participants completed during the experiment. The bilingual participants tested by Wu and Thierry were instructed to make rhyme judgments for picture pairs. This task required participants to focus on the linguistic relationship between the prime and target pictures and narrow their focus to one language in order to successfully complete the task. When tested in L1, participants were better at narrowing their focus and preventing interference from L2, but this was not the case when participants were test in L2 and as a result L1 words were also activated. In the current study, bilingual participants judged whether the picture prime and a subsequently presented picture (after the target word) were the same or different. This task did not require participants to pay attention to the relationship between picture prime and target word. Unlike the participants tested by Wu and Thierry, the participants tested in the current study did not need to narrow their focus to one language in order to successfully complete their task. We suggest that this difference in task is the reason we find implicit label generation in both languages when participants were tested in L1, while Wu and Thierry did not. It is especially useful for future research that implicit label generation in bilinguals can be studied without using a task that calls attention to the relationship between prime and target.

We note that our results also contrast with previous work by Weber and Cutler (2004) who report that bilinguals do not activate their L2 in L1 processing, when immersed in an L1 dominant environment. In our study, similar to Weber and Cutler (2004), participants were immersed in their L1 and tested in their L1. In this situation, the only relevant language is L1. Yet, our results demonstrate that participants implicitly generated the label for prime pictures in both L1 and L2. One possible explanation is the potential difference in L1 and L2 use between the Dutch-English bilinguals tested by Weber and Cutler, and the German-English bilinguals tested in the current study. Previous studies have found that an L2 immersion context has a positive influence on L2 proficiency and performance compared to L2 classroom exposure, although this comes at the cost of L1 fluency (Linck et al., 2009; see also Baus et al., 2013). Faced with the task of L2 usage every day, bilinguals may inhibit their L1 (Green, 1998) in order to perform successfully in their L1 or this may simply be the result of reduced frequency of L1 use (Gollan et al., 2005, 2008). In the context of L1 immersion, L2 fluency may also experience a reduction, which would account for the findings of Weber and Cutler but not those of the current study. The difference, then, would lie in the usage of L2 English in the different L1 contexts of Dutch and English. Although comparisons of English proficiency and frequency of use across cultures are at the moment anecdotal at best, such considerations are crucial to the future study of bilingual language processing. Alternatively, it is possible that the use of a more sensitive paradigm to assess participants' access to L2 words, i.e., the cross-modal ERP priming paradigm, may have allowed us to tap into cross-language effects that could not be observed in Weber and Cutler. Indeed, a number of studies have shown that such subtle cross-language effects do not lead to noticeable differences in responding while triggering different patterns of neural activity (Kotz and Elston-Güttler, 2004; McLaughlin et al., 2004; Tokowicz and MacWhinney, 2005; Thierry and Wu, 2007; Wu and Thierry, 2010a; for a review see Mueller, 2005).

We suggest that, while future studies are needed to compare different language combinations and environments, the cross-modal ERP priming paradigm used in the current study is useful tool for measuring bilingual co-activation and allows us to tap into subtle effects of other language activation in bilingual language processing. This finding, taken together with other studies using different methods (Spivey and Marian, 1999; Wu and Thierry, 2011; Von Holzen and Mani, 2012) suggests that activation of both languages during processing in one language is a powerful phenomenon. While viewing pictures, bilinguals activate the corresponding label as well as its phonological information in both of their languages. Although such a result cannot be generalized outside of an experimental setting, it affords a glimpse into the complex processes that bilinguals undergo while interacting with their environment.

### Cross-language priming in an L1 experimental setting

The current study also provides evidence for cross-language priming in bilinguals such that implicitly generated L2 labels facilitate recognition of auditory L1 targets despite the experiment being conducted entirely in one language. Although relatively unexplored in auditory word processing (but see Phillips et al., 2006; Pratt et al., 2013), previous studies have also found similar L2-L1 priming effects for phonologically related prime-target pairs in visual word processing (Van Wijnendaele and Brysbaert, 2002; Duyck, 2005; Zhou et al., 2010). These studies, however, used both languages in their experiments, and priming effects may, therefore, result from the artificial bilingual environment created by using both languages in the experimental setting (Grosjean, 1997). In contrast, the current study was conducted in one language. The between-language priming effect, therefore, cannot be attributed to an artificial bilingual environmental setting and the current results present the first evidence for L2-L1 priming in auditory word processing in an unbiased setting. This a useful tool for future studies to continue studying cross-language phono-lexical effects in bilinguals without presenting word stimuli auditorily or visually in both languages.

### Implications for models of lexical access during speech production

The two main findings of the current study, namely that bilinguals implicitly generate the labels for pictures in both of their languages and cross-language phonological priming in participants immersed in their L1, provide an interesting addition to the debate on the kind of information that is activated during word production. It is generally accepted that speech production involves first activating the lexical node associated with the concept, followed by the corresponding phonological code (Levelt, 1989; Roelofs, 1992; Caramazza, 1997; Dell et al., 1997). Conflict abounds, however, with regard to whether the phonological information of non-selected lexical nodes is also activated. Discrete serial models of lexical access suggest that only the phonological information associated with the selected lexical node is activated (Levelt, 1989; Levelt et al., 1999). For example, when naming a picture of a *table*, semantically related lexical nodes are also activated (i.e., *couch*, *chair*). Ultimately, the lexical node for *table* is selected and the corresponding phonological information is activated, but not for the non-selected lexical nodes (i.e., *couch*, *chair*). Cascaded activation models of lexical access, in contrast, propose that the phonological information from both the selected and non-selected lexical nodes is activated (Dell, 1986; Caramazza, 1997; Dell et al., 1997).

The findings of the current study and Wu and Thierry (2011), provide useful information with regard to the processes underlying picture naming and, by extension, the extent to which conceptual and phonological levels of representations are recruited in speaking. Neither of these studies used prime-target pairs with semantic overlap (although Wu and Thierry did include a semantically related condition, prime-target pairs in the critical rhyming conditions were not additionally semantically related). Nevertheless, both studies demonstrate that participants activate phonological information associated with non-selected lexical nodes in implicit generation of the labels for visually fixated images. In other words, participants activated phonological information associated with both L1 and L2 labels for the images. If purely semantic information associated with non-selected lexical nodes were activated, as suggested by discrete serial models (Levelt, 1989; Levelt et al., 1999), we would have expected priming effects only in the identity condition. But, the priming effect for the between-language condition in the current study provides support for cascaded activation models of lexical access (Dell, 1986; Caramazza, 1997; Dell et al., 1997), by demonstrating that phonological information from both selected (L1) and non-selected (L2) lexical nodes was activated when our participant viewed the prime pictures. Our study, furthermore, goes beyond Wu and Thierry (2011) in showing that participants activated L2 labels for the prime images despite being immersed in their L1 and tested in their L1, thereby reducing the likelihood that L2 lexical nodes would need to be retrieved.

### Conceptual activation during implicit label generation in bilinguals

The results of the current study have interesting implications for models of bilingual speech processing with regard to the activation of language representations from conceptual representations during picture viewing. The Revised Hierarchical Model is a model of bilingual word production that focuses on the connections between L1 and L2 words at the lexical and conceptual levels and how these connections develop as proficiency increases (Kroll and Stewart, 1994; Kroll and Dijkstra, 2002; Kroll et al., 2010). According to this model, connections exist between L1 and L2 translation equivalents at the lexical level. At the conceptual level, however, connections initially exist only between L1 words and their concepts. Access to conceptual representations during L2 processing must, therefore, be mediated through L1 translation equivalents. With greater L2 proficiency, access to conceptual representations during L2 processing may continue without mediation through L1 translation equivalents, with direct links between L2 words and their conceptual representations.

Models of speech production argue that upon viewing a picture, participants activate the conceptual representations associated with this image, leading to phonological activation of either one or many selected lexical nodes associated with the activated conceptual representations. Thus, evidence of the activation of the L2 label for the image might be taken to suggest that, in proficient bilinguals, conceptual representations activated are directly linked to L2 labels such that viewing the picture leads to activation of conceptual representations which in turn directly activate both L1 and L2 labels. Alternatively, it is possible that, even in proficient bilinguals, L2 words are only indirectly linked to conceptual representations such that the results of the current study are explained as follows: viewing the picture leads to activation of conceptual representations, which in turn activate the L1 label, leading to mediated activation of the L2 label from the L1 label. While our study cannot rule out this explanation entirely, we note that there were no differences in the time-course or strength of the effects across the within-language and between-language overlap conditions, suggesting that such mediated activation through L1 labels is unlikely. To this extent, the results of the current study support the suggestion of the RHM that, in proficient bilinguals, L2 words are directly linked to their concepts.

Alternatively, we note that the results could also be explained without relying on access to the conceptual level. For example, the visual features of a picture may directly activate its' label, such that, upon viewing a picture, the visual features of the picture activate the corresponding labels in both languages. Activation of word labels from picture viewing relies, in this case, not on the activation of conceptual representations but rather on the recognition of the visual features of an image. It will be interesting, therefore, for future studies to explore the extent to which conceptual representations are involved in the progression from image recognition to implicit naming/production. We also note that regardless of whether L2 labels were activated directly from conceptual representations or from the L1 labels, the results of the current study strongly suggest that bilinguals implicitly produce the labels for visually fixated images in both their languages, even when immersed in an L1 setting and when tested in their dominant language.

## Conclusion

The current study presented evidence that bilinguals implicitly generate the label for pictures in both of their languages. Previous studies have shown mixed results, suggesting that an L1 language environment (visual world paradigm; Weber and Cutler, 2004) or experimental task that requires participants to focus on the linguistic relationship between prime and target (rhyming judgment task; Wu and Thierry, 2011) may prevent co-activation of L2 words. By using a cross-modal ERP priming paradigm, we demonstrated not only that bilinguals implicitly generate the label for pictures in both of their languages, but also that implicitly generated L2 labels can prime related L1 words. The results provide support for cascaded activation models of lexical access, showing that phonological information associated with non-selected lexical nodes is retrieved during (implicit) picture naming.

### Conflict of interest statement

The authors declare that the research was conducted in the absence of any commercial or financial relationships that could be construed as a potential conflict of interest.

## Footnotes

^1^As complete phonological overlap between words across languages rarely occurs (Dijkstra et al., 1999), reducing our stimuli set to prime-target pairs that overlap completely across languages would have severely reduced our word choice for prime-target pairs for the between-language related condition. As a result, we chose prime-target pairs that sounded similar to one another in English and German, respectively.

^2^An analysis on the data without removing trials that were produced correctly showed the same pattern of results as the analysis reported.
