# Supplementary material for: Bilinguals implicitly name objects in both their languages: an ERP study
Source: Front Psychol. 2014 Dec 9;5:1415. doi: 10.3389/fpsyg.2014.01415 (PMC4260478; doi:10.3389/fpsyg.2014.01415)
Supplement: Supplementary file 1 [file DataSheet1.PDF]

Appendix A

List of stimuli presented to participants, separated by condition. English and German labels for prime pictures as well as the German target words and their English translations are given.

	Prime pictures		Target word	
Condition	English label	German label	German	English translation
Identical	ant	Ameise	Ameise	ant
	basket	Korb	Korb	basket
	beach	Strand	Strand	beach
	bee	Biene	Biene	bee
	bird	Vogel	Vogel	bird
	candle	Kerze	Kerze	candle
	cat	Katze	Katze	cat
	cherry	Kirsche	Kirsche	cherry
	cup	Tasse	Tasse	cup
	fiddle	Geige	Geige	fiddle
	glasses	Brille	Brille	glasses
	heart	Herz	Herz	heart
	key	Schlüssel	Schlüssel	key
	lion	Löwe	Löwe	lion
	milk	Milch	Milch	milk
	monkey	Affe	Affe	monkey
	moon	Mond	Mond	moon
	ostrich	Strauß	Strauß	ostrich
	pear	Birne	Birne	pear
	rain	Regen	Regen	rain
	reel	Spule	Spule	reel

	saw	Säge	Säge	saw
	shirt	Hemd	Hemd	shirt
	snake	Schlange	Schlange	snake
	spoon	Löffel	Löffel	spoon
	star	Stern	Stern	star
	top	Kreisel	Kreisel	top
	tree	Baum	Baum	tree
	water	Wasser	Wasser	water
	window	Fenster	Fenster	window
Within-language	bone	Knochen	Wochen	weeks
	book	Buch	Tuch	towel
	bottle	Flasche	Tasche	bag
	box	Kasten	Masten	poles
	button	Knopf	Topf	pot
	card	Karte	Warte	look-out
	comb	Kamm	Schwamm	sponge
	crane	Kran	Hahn	cock
	dog	Hund	Mund	mouth
	doll	Puppe	Suppe	soup
	earth	Erde	Herde	flock
	flag	Fahne	Sahne	cream
	fox	Fuchs	Luchs	lynx
	gate	Tor	Chor	choir
	goat	Ziege	Wiege	cradle
	grape	Traube	Schraube	screw

	juice	Saft	Kraft	strength
	mountain	Berg	Zwerg	dwarf
	mug	Becher	Sprecher	speaker
	nun	Nonne	Sonne	sun
	pan	Pfanne	Tanne	christmas tree
	pants	Hose	Dose	can
	pig	Schwein	Bein	leg
	pigeon	Taube	Glaube	belief
	straw	Stroh	Floh	flea
	switch	Schalter	Falter	butterfly
	tooth	Zahn	Bahn	railroad
	whale	Wal	Schal	scarf
	whistle	Pfeife	Seife	soap
	worm	Wurm	Turm	tower
Between-language	beagle	Spürhund	Ziegel	brick
	belt	Gürtel	Geld	money
	car	Auto	Schar	cohort
	cow	Kuh	Stau	traffic jam
	cross	Kreuz	Schloss	castle
	crown	Krone	Zaun	fence
	door	Tür	Ohr	ear
	duck	Ente	Lack	lacquer
	feet	Füsse	Riet	reed
	file	Feile	Beil	ax
	fire	Feuer	Eier	eggs

	kite	Drachen	Zeit	time
	kitten	Kätzchen	Sitten	manners
	knight	Ritter	Leid	sorrow
	leaf	Blatt	Tief	deep
	neck	Nacken	Scheck	check
	pea	Erbse	Knie	knee
	peel	Schale	Ziel	goal
	quilt	Steppdecke	Wild	venison
	raft	Floß	Schaft	sheath
	sheep	Schaf	Dieb	thief
	slide	Rutsche	Kleid	dress
	snout	Schnauze	Braut	bride
	spider	Spinne	Neider	envier
	spine	Wirbelsäule	Stein	rock
	suit	Anzug	Blut	blood
	tire	Reifen	Schleier	fog
	tower	Turm	Bauer	builder
	truck	Laster	Frack	dress coat
	wheel	Rad	Spiel	game
Unrelated	bench	Bank	Siedler	settler
	blackboard	Tafel	Pendel	pendulum
	boot	Stiefel	Wetter	weather
	bread	Brot	Wolken	cloud
	bullet	Kugel	Ecke	corner
	cage	Käfig	Haare	hair
	candy	Bonbon	Münze	coin

cheese	Käse	Bau	building
chicken	Huhn	Hafen	harbor
church	Kirche	Schilf	reed
deer	Hirsch	Linie	line
dice	Würfel	Körper	body
doctor	Arzt	Rede	speech
eye	Auge	Müll	garbage
handle	Griff	Saal	hall
pitcher	Krug	Welle	wave
knife	Messer	Seil	rope
lightning	Blitz	Aal	eel
nail	Nagel	König	king
pillow	Kissen	Vieh	cattle
rhino	Nashorn	Muskel	muscle
sausage	Wurst	Kehle	throat
stool	Hocker	Stoff	stuff
suitcase	Koffer	Szene	scene
swan	Schwan	Hüfte	hip
tent	Zelt	Beleg	evidence
thorn	Dorn	Kalb	calf
thumb	Daumen	Schacht	pit
toe	Zeh	Kuppel	dome
vulture	Geier	Traum	dream
